# Enhancing national progress and sustainable economic development among *Al-Majiri* children in Northern Nigeria

**DOI:** 10.1016/j.heliyon.2021.e08066

**Published:** 2021-09-23

**Authors:** Yusuff Jelili Amuda

**Affiliations:** College of Law, Prince Sultan University, Riyadh, Saudi Arabia

**Keywords:** *Al-Majiri*, National progress, Sustainable economic development, UBEC, NBAIS

## Abstract

*Al-Majiri* is an itinerant or traditional Islamic educational system commonly known in the Northern part of Nigeria for cultivating spirituality and inculcating morality prior to colonialism. The prevalent street begging by the *Al-Majiri* children has not contributed to sustainable economic development because of lack of vocational training and entrepreneurships which remains a big gap to fill in the existing body of knowledge. The primary objective of this article therefore, is to investigate vocational training and entrepreneurship skills towards improving *Al-Majiri* system for attaining national progress and sustainable economic development in the country. Narrative-textual case study and systematic literature review (SLR) were employed as the methodology of the paper where different sources of problem are identified and solutions are proffered for addressing the identified problems. The findings indicated that, inclusion of vocational training and entrepreneurship skills into *Al-Majiri* system such as shoe-making, tailoring, mechanics, poultry farming, repair of phones, weaving among others can contribute to national progress and sustainable economic development. In conclusion, it is said that, the system of *Al-Majiri* can be improved, if necessary, mechanisms are being put in place to eradicate illiteracy, inequality and poverty among the students. It is therefore suggested that, all the stakeholders namely: government, school administrators, parents, teachers, students, individuals and society should collaborate in putting effective measures in place for future educational efficiency of *Al-Majiris* in the country.

## 1 Introduction

The role of education in driving socio-economic development in a given nation cannot be underestimated. At the global level, education is considered as a tool for human resource development. This is significant because knowledge driven economic at the global level is highly competitive. Education is indispensable in the discourse of development. Man is the centre of education because he is the one that contributes to human and material developments of the society. Education is very important in the discourse of overall development of learners especially in improving their thinking, attitudes and skills. According to Andy (2007), education gives individual opportunity to growth according to his/her ability within his society especially by acquiring necessary skills in order to enable him feed himself and his family and subsequently makes him contribute meaningfully to the society in general. There are various indices of development such as: education, social, economy, infrastructure among others (Bassey, 2011). Indeed, education is an essential indicator of development because it is the vehicle that drives other components of development (Daramola, 2010).

Education has been a major determinant for human and material resources. The education of *Al-Majiri* system is given less concern in this regard despite the fact that, the population of the total number of ten million (Ministerial Committee on Madrasah Education, 2010; UBEC, 2010) in different parts of the North. *Al-Majiri*, Almajirci or Almajiranci is a Hausa word emanated from Arabic word of Al-Muhajirun which means emigrants (Bala, 2018). It technically refers to those that migrate from their homes for the purpose of acquiring knowledge (Imam, 2015). Literature further contends that, there is need for the integration between religious and non-religious subjects in *Al-Majiri* system in the country (Ahmad and Awang Mat, 2011). The workability of the system is that, it only focuses on religious learning and lack of essential vocational training that can contribute to sustainable socio-economic development in the country. *Al-Majiri* children as a result of poverty are affected in contributing to the socio-economic development of the country. Literature such as Akhakpe (2009) posits that poverty hinders sustainable socio-economic development. In addition, these *Al-Majiri* children are being used for electoral violence and they are not isolated from other criminal activities that are prevalent in the society (Yusha'u et al., 2013). All these concerns need proactiveness in finding an effective solution and sustainable means in addressing this challenge. In so doing, since education is considered significant for human and national development, *Al-Majiri* children are not adequately being prepared for the future employment.

It is not disagreeable to posit that, the educational system of *Al-Majiri* in the Northern part of Nigeria has not manifested essential indices of development. This problem is not only restricted to *Al-Maijiri* but the entire educational enterprise of the country has not demonstrated sufficient and expected development in the country. There are technical skills required for the overall development of the country and these skills are lacking among *Al-Majiri* children in order to achieve national and sustainable development (Isiaka, 2015). The large number of *Al-Majiri* children are found incapable of doing certain works but this is not because they do not have capabilities but because opportunities are not being given to them to utilize their potentials. This problem continues to surface as a result of the fact that, *Al-Majiris* have not been connected with industrial and socio-economic needs of the society (Bala, 2018). It is not arguable to say that, the competence of some of these *Al-Majiri* children need to be cultivated but there has been undue negligence in their part. Hence, adequate focus should be given to improvement of social and economic conditions of destitute *Al-Majiri* children in the Northern part of the country.

Onwards, literature contends that, the system has not contributed to socio-economic and infrastructural development of the country as a result of the fact that, there is no meaningful attention given to what is happening in *Al-Majiri* system as part of strategy for attaining national progress and sustainable development (Imam, 2015; Bala, 2018). Nonetheless, the character building is an essential element that manifests the focus of the system. This has been acknowledged in the literature that, religious moral education contributes significantly to sustainable development in Africa (Akwaki and Jamberlang, 2020). However, today, the system cannot boldly claim the character building because of the allegation that the system produces destitute who cannot offer meaningful contributions to the society. In other words, the character building inherent with the system remains questionable because the system does not cater for livelihood of the teachers (*malams*) and the students (*Al-Majiris*) which make them to be considered as people living below the standard which the study of Bala (2018) has emphatically stressed on the incorporation of vocational education into the system. Education is all about developing human potentials that will transform positively in order to make worthy living for everyone. Education is not trade, it is not business but it is an important element in propelling trade and business activities which is an important climax of sustainable development in the society (Ajala, 2007).

More so, pertaining to dialogue or debate relating to development in the modern time, functional education remains pivotal to the discourse of sustainable development. Hence, there is need for redirection of educational focus in the context of *Al-Majiri* system specifically in portraying it to contribute to socio-economic and sustainable development in the country. It is thereby important that educational system of *Al-Majiri* can be made sustainable if essential skills such as entrepreneurship skill are incorporated into the overall activities of the system (Imam, 2015). However, literature identifies that a number of factors inhibit sustainable development and of such factors are: high rate of poverty, social exclusion, mismanagement of national economy and poor educational development (Akhakpe, 2009; Daramola, 2010). Undeniably, the aforementioned factors are true about what *Al-Majiri* system is facing in various Northern states. For instance, *Al-Majiri* children with experience of street begging is an indicator of determining high rate of poverty. As a result of negligence in the part of these children, they experience social exclusion consequently which made them to indulge in social vices.

Moreover, the inadequate educational system of *Al-Majiri* requires adequate policy that will include these children into the core focus of national development. Similarly, misplacement of priority manifesting from mismanagement of national economy immensely contributes to the overall challenges facing *Al-Majiri* in the North. Economic growth is important for sustainable development whereby education immensely contributes to it. Thus, meaningful development can be attained if educational system of *Al-Majiri* is reviewed alongside with provision of entrepreneurial activities that will boast investments and business enterprise which will enormously contribute to socio-economic development of the country. It is only by this means that, negative impression about *Al-Majiri* can be changed for better practice. This can be changed for better specifically by making the overall system sustainable in the country. There is a connection between education and socio-economic development. Without proper skills like entrepreneurship skill, *Al-Majiri* children can become a liability to the society and to the entire country as the issue of Boko Haram has directly linked with the system of *Al-Majiri* in the Northern Nigeria. It is therefore important to explore sustainable educational development that will prepare *Al-Majiri* students for the future especially in meeting up with the knowledge driven world in order to achieve sustainable development in the Northern part in particular and the entire country in general.

## 2 Methodology

This part explains the research design, data collection and data analysis. The article used narrative-textual case study is employed as the methodology of the article where different sources of problem are identified and solutions are proffered for addressing the identified problems. In addition, the secondary data was obtained through the use of systematic literature review (SLR) of the existing body of knowledge (Ministerial Committee on Madrasah Education, 2010; Abdulazeez and Musa, 2015). Literature posits that, SLR is purposely used in order to clary ideas (Pittaway and Cope, 2006). More importantly, the secondary data used in this paper was based on the what was obtained from the study of which was accessed through Internet. Thus, there are five Northern states chosen for the study namely: Kano, Sokoto, Kaduna, Niger and Borno. The population of *Al-Majiri* students in the selected states was estimated as 4,493, 200. Indeed, there was no statistical tests used in the paper because there is a complete reliance on the secondary data for the period of five years (i.e., 2015–2020) as well as the use of systematic literature review (SLR). Based on the use descriptive narrative design of the study, paradigmatic case was used selected in order to address a phenomenon by revealing key elements of that particular phenomenon. there were three major themes generated from the phenomenon of *Al-Majiri* namely: Statistical figures of *Al-Majiri* System, *Al-Majiri* Educational System for Sustainable Development and Sustainable Economic Development among *Al-Majiri* Children. Thirty-two scholarly articles were exhausted in this study. Hence, pragmatic mode of analysis of the aforementioned themes was used. The subsequent subheadings present the results of the paper.

## 3 Results and discussion of major findings

This section presents the results of the paper based on systematic literature review and analysis of secondary data obtained from the existing literature (Ifijeh and James (2015). The results are discussion of major findings were done in accordance with the four themes generated namely: Conceptualization of Sustainable Development, Statistical figures of *Al-Majiri* System, *Al-Majiri* Educational System for Sustainable Development and Sustainable Economic Development among *Al-Majiri* Children. Each of these is presented in the subsequent sub-headings.

## 4 Conceptualization of sustainable development

This part explains the conceptualization of sustainable development in connection with of *Al-Majiri* system in the Northern part of Nigeria. The discourse on sustainable development at the international level started after Rio Earth Summit in 1992. It was United Nations Conference on Human Environment that provided the framework for its operation. Undoubtedly, literature identifies that education plays a significant impact in prevention ecological degradation (Cleveland and Kubiswezki, 2007). Literature posits that, World Conservation Union as manifested in the strategy used to popularize the term sustainable development (Adebayo, 2010). It is further asserted that, sustainable development is an advocacy for a change in social structure and attitude which brings about acceleration of economic growth by commitment to reduction of social injustice, abject poverty and inequality in the society (Adebayo, 2010). The study by Sachs (2012) presents sustainable development according to the World Commission on Environment and Development (WCED) is, “development that meets the needs of the present situation without comprising the ability of the future generation to meet their own needs”. This is important and relevant to the needs of *Al-Majiris* in the North because the current practice has compromised the ability of the children which deny them in meeting their own needs as well as fulfilling the needs of the whole nation. This is so because essential skills are not incorporated into the system.

Thus, there is need to change the pattern of the current system in order to attain sustainable development of the system. It can be asserted that, sustainable development is regarded effort towards improving socio-economic aspect of the society by galvanizing various resources together in order to enhance quality of life (Adebayo, 2010). More specifically, Mohammed as cited in Adebayo (2010) relates sustainable development as:

Increasing the availability and widening the distribution of basic life sustenance such as food, shelter and protection; raising the level of living in addition to higher income, provision of jobs, better education and greater attention to culture and humanitarian values, all of which serve to enhance material well-being, but also to generate greater individual and national self-esteem; and expanding the range of economic and social choice to individual and nations by freeing them from servitude and dependence (p.214).

It is indisputable to say that, for *Al-Majiri* system to be sustainable, education is an essential tool to achieve this. Thus, it is important to improve their socio-economic condition especially by providing business opportunities for *Al-Majiri* children through the establishment of entrepreneurship skill in order to independently raise the level of their income after graduation. More specifically, the concept is related to three major aspects of human wellbeing namely: economic wellbeing, environmental sustainability and social inclusion. According to Sachs (2012), sustainable development in connection with the aforementioned factors differs from one country to the other. However, the triadic components are considered as broad consensus at the international level. Nevertheless, economic wellbeing, environmental sustainability and social inclusion are paramount factors for attaining overall development in *Al-Majiri* system in the Northern part of the country. It should be stressed that, education remains viable mechanism in fighting poverty and underdevelopment as predominant with *Al-Majiri* system in the Northern part of the country. It is thereby important to improve the condition of *Al-Majiris* in order to attain sustainable development in the country.

## 5 Statistical figures on Almajiri system

This part presents the statistical figures on Almajiri system in order to demonstrate the vital importance of integration of knowledge for national and socioeconomic development in the country. Undoubtedly, the studies such as Abdulazeez and Musa (2015) posit that the Federal government of Nigeria showed interest in enhancing *Al-Majiri* system into mainstream educational system in the country.

The integration of *Al-Majiri* system was extensively discussed at the National Economic Council in 2013 and National Committee on the Implementation of *Al-Majiri* Education Programme was set up with specific target of ensuring that children of *Al-Majiri* system are provided with opportunities to have access to Basic Education. Statistical data by the Ministry of Education shows that, there are total numbers of 9.5 million *Al-Majiri* children that attend traditional Islamic schools in the Northern Nigeria (Ministerial Committee on Madrasah Education, 2010).

More specifically, an approximate of 300, 000 almajiri children are residing in Kano. Nonetheless, other studies such as Ifijeh and James (2015) contend that, there are few states with predominant *Al-Majiri* children. It is further asserted by Ifijeh and James (2015) that, the entire Kano state harbors 1.6 million *Al-Majiri* children in total number 26, 000 madrasahs; Sokoto with approximate of 1.1 million; Kaduna, 824, 200; Niger with 580, 000 and Borno with 389, 000 *Al-Majiri* children respectively. Table 1 shows selected states with statistical figure of Almajiri Population:

**Table 1:** Selected states with statistical Figure of Almajiri population.

S/N	Selected States with Predominant *Al-Majiri* Children	Statistical Figure of *Al-Majiri* Population
1.	Kano State	1.6 Million
2.	Sokoto State	1.1 Million
3.	Kaduna State	824, 200
4.	Niger	580, 000
5.	Borno	389, 000
	**Total =**	**4,493, 200**

***Source:*** Ifijeh and James, 2015

The forgoing has demonstrated the statistical figure of Almajiri population in the Northern part of the country. Nonetheless, the system has been criticized for promoting delinquency and poverty as a result of lack of vocational skills that will prepare them for workforce (Taiwo, 2013; Isiaka, 2015). These children need to be provided with skills that will enhance socio-economic development in the country which could be considered as prospects for the system (Yusha'u et al., 2013). Hence, a total number of 125 *Al-Majiri* model schools were built in 27 states across the country. More specifically, the Universal Basic Education Commission (UBEC) built 89 schools while Tertiary Education Trust Fund (TetFund) constructed 64 schools. Additionally, literature posits that, the schools were jointly funded by the Islamic Development Bank (IDB) and the Nigerian government with the sum of $98 million (Omeni, 2015). The constructed schools were handed over to the state government. It is noted that, there are three major elements required in the integration of knowledge which literacy, numeracy and life skills. It should be stressed that life skills are directly linked with enhancement of socioeconomic development in the country. More specifically, it is noted that, life skill subjects to be harmonized with other subjects in order to foster socioeconomic development are: Agriculture, commerce, trade, handcraft, mechanic, vulcanizing and many other vocational skills (Abdulazeez and Musa, 2015; Isiaka, 2015).

## 6 Enhancing *Al-Majiri* educational system for sustainable development

At the international level, there was Declaration of the World Conference on Education for All (WCEFA) which was held at Jomtien, Thailand in 1990. The prime target of the conference was to ensure that citizens from various countries have opportunities to be educated in order to fulfill his/her basic needs. Notably, the United Nations have been advocating for Sustainable Development Goals (SDGs) which is essential for the country to provide opportunities for the citizens in order to achieve the overall progress. It is however unfortunate that, the current educational system in *Al-Majiri* system in Nigeria does not prepare students to be part of human capital development which will make the students contribute to the nation's innovation and socio-economic competitiveness. In response to the above, the philosophy of education in the context of Nigeria reflected in National Policy on Education (NPE) mentions essential points among which is to make, ‘education fosters the worth and development of the individual, for each individual's sake and for the general development of the society’. More specifically, it is stressed in the NPE (2013) that:

Education in Nigeria is an instrument “par excellence” for effecting national development…. It is therefore desirable for the Nation to spell out in clear and unequivocal terms the philosophy and objectives that underlie its investment in education…for the benefits of the citizen… to the needs of the individuals… its consonance with the realities of our environment and the modern world (p.2)

As part of pursuance of the Education for All and attainment of this educational philosophy, the federal government of Nigeria established Universal Basic Education Commission (UBEC) in order to eradicate illiteracy, poverty and inequality in the country (Ker, 2006; UBEC, 2010). The government has been trying in this regard by including *Al-Majiri* system into UBEC especially by harmonizing conventional subjects together with Islamic subjects which is an idea of addressing the challenge of poverty and illiteracy among *Al-Majiri* children (Ahmad and Awang Mat, 2011).

Onwards, National Board for Arabic and Islamic Studies (NBAIS) which had been in existence since 1960 had been playing significant role in improving the system of *Al-Majiri* specifically in the area of modernization of the system. The Law No.10 of the then Northern Nigeria established the NBAIS and it had a centre at Arewa house in Kaduna. It was later moved to the Institute of Education, Ahmadu Bello University, Zaria after the creation of states from the defunct Northern Region (Adelani, 2020). Since few years ago, the federal government of Nigeria has given recognition to NBAIS attaining the same status with two major national examination bodies namely: West African Examination Council (WAEC) and National Examination Council (NECO) (Ibrahim and Abdur-Rafiu, 2020). The main responsibility of NBAIS is to coordinate and standardize the curriculum of *Al-Majiri* and *Islamiyyah* schools in different parts of the country (Adelani, 2020). However, there is still need to put more effort especially in integrating entrepreneurship skill into the programme in order to make them useful to themselves and the society at large.

With growing concern by different agencies and authorities in addressing the problem of *Al*-*Majiri* system in the Northern part of the country, it is thus important that despite the fact that, the paper has clamoured for inclusion of entrepreneurship skills, it is necessary to transform the system into viable or sustainable development in order to drastically reduce poverty in the society (Iwala, 2014). It is noteworthy to say that, there is no unanimous definition of the term; but different scholars and experts examine the concept in different ways. According to Omare (1999), Sustainable development deals with policy formulation and implementation by the Government in order to provide programmes that will be useful to the present generation as well as future generation especially focusing on an everlasting solution to multifarious challenges of the society. Similarly, literature contends that, sustainable development refers to an ability to live within the capacity of a group of people without compromising the future needs of the younger generation (Kuhlman and Farrington, 2010). More importantly, literature contends that, sustainable development can be regarded as a system of governance whereby government attempts to provide necessary amenities and maintain resources which will make their lives conducive for the contemporary needs without compromising the needs of future generation (Erhun, 2015; Oludare, 2020).

Based on this, it can be inferred that, in the past, necessary resources were not being provided to *Al-Majiri* children by the government in order to cater for their future. Nevertheless, in the recent, the government has been making tremendous effort in ensuring that the system is sustainable in enhancing the capacity of *Al-Majiri* children with specific attention on their social needs and socio-economic development (Imam, 2015; Bala, 2018). In order to achieve this, sustainable development is discussed from the perspectives of policy transformation, lifelong learning for business venture, internalization of religious values, structural, institutional and attitudinal changes and sense of patriotism (Dang and Sui-Pheng, 2015).

Furthermore, sustainable development can be achieved in the context of *Al-Majiri* system when entrepreneurship skill and vocational training are emphasized as part of policy that will transform it into viable economic hub in the Northern region as literature contends (Bala, 2018). This can also be achieved when there is a responsible leadership which provides resources and amenities to the underprivileged *Al-Majiri* children. It is not doubtful to say that *Al-Majiri* has been trying in teaching religious knowledge and cultivating character but it remains a plethora social problem especially in ensuring that street begging of these innocent children is addressed (Isiaka, 2015). Hence, entrepreneurship skill can be considered as a transformation of *Al-Majiri* in contributing to socio-economic development of the country as several studies have clamoured for diversification of economic in attaining sustainable growth and economic development (Suberu et al., 2015).

The current scenario in *Al-Majiri* system does not reflect sustainable development because teaching and learning does not transform into appreciable level of making it to cultivate the potential of students to contribute to inclusion of vocational training and entrepreneurship skill for attaining socio-economic growth through their active participation in overall development of the society (Imam, 2015). In order to achieve the overall development, it is important to make religious education through teaching changes the value and perception about life. Notably, *Al-Majiri* system can be seen as being sustainable when learning is regarded as lifelong process which provides an avenue for the learners to acquire creative thinking, problem solving and decision-making skills that will be instrumental in propelling vocational training and entrepreneurship activities among the students (Oladosu, 2012).

It is reiterated that; sustainable development should be contextualized in the *Al-Majiri* system in the Northern part of the country by providing opportunities such as entrepreneurship skill. This can only be achieved by activating the aspirations and potentials of *Al-Majiri* children especially by involving them in entrepreneurial activities. This inferably means that, there is paramount relationship between sustainable development and entrepreneurship activities in improving socio-economic growth among *Al-Majiri* children. Sustainable development can be achieved with an emphasis business venture that will determine the economic growth of the nation. In other words, entrepreneurship skill that is expected to be cultivated in children is considered as catalyst for sustainable socio-economic of the society.

Furthermore, it should be said that, since violence and many other social vices have been attributed to *Al-Majiri* system in the North, it is important to de-radicalize the whole system by emphasizing of on the internalization of religious values such as love, respect, tolerance etc. that will make the *Al-Majiri* attain moral development alongside sustainable development. It is on this basis that literature asserts that, violent conflicts disrupt the process of production, creates condition for pilferage of the country's resources and diverts their application from development purpose to servicing war (Iwala, 2014).

The abovementioned position is correct in the context of *Al-Majiri* system because allowing these children to partake in the street begging can make them fall prey of involving in atrocities such as bombing which will surely affect the process of production as an integral part of entrepreneurial activities that this paper advocates for. Hence, proper transformation of *Al-Majiri* system can prepare students for the contemporary educational challenges among Muslims in the Northern Nigeria. In order to achieve the foregoing, there is need for collaborative effort between the government and owners or proprietors of these *Al-Majiri* schools towards sustainable development of the system. It is in this regard that literature contends that, in order to achieve sustainable development, there is need to put in place structural, institutional and attitudinal changes for religious education (Oludare, 2020).

This is significantly important in the context of *Al-Majiri* schools because government has been putting structural change in place through the establishment of *Al-Majiri* Integrated Model Schools (AIMS) in various places in the Northern part of country. Regarding institutional change, the proprietors of private *Al-Majiri* schools are expected to cooperate with the government policies as well as to maintain facilities that are being given for the improvement of the system. Pertaining to attitudinal change, all the stakeholders such as government, proprietors, teachers and parents have vital roles to place specifically in changing the street begging commonly known with the *Al-Majiri* students in the North. Also, the government is expected to address socio-economic injustice and inequality that affect *Al-Majiri* children by providing entrepreneurship activities which will make them self-reliant. In order for the government to address the above, literature contends that, religious communities and leaders are expected to raise their voices in addressing social inequality and economic injustice especially towards transformation of *Al-Majiri* system for the benefit of the society (Isiaka, 2015). More importantly, social inequality and predominant economic injustice in, *Al-Majiri* system should be addressed.

Moreover, another issue that has to do with the sustainable development is the sense of patriotism. Patriotism refers to the sense of selfless service and commitment to one's nation. It is not disagreeable to assert that, *Al-Majiri* children are not patriotic because it is not cultivated in them. This perception should be changed and the sense of patriotism should be cultivated in learners of *Al-Majiri* system in order to attain substantial level of sustainable development (Bala, 2018). Thereby, there is need to change negative impression of regarding *Al-Majiri* inimical to the overall sustainable development by making it viable for entrepreneurial activities. It can be reiterated that, if sustainable development should be achieved in the country, attention should be given to the social and economic conditions of *Al-Majiri* children especially by incorporating vocational training into the system (Bala, 2018).

Leadership is a vital factor in determining good governance. In other words, good governance cannot be achieved without adequate and effective leadership. Leadership and good governance are important elements in building structures and social institutions that can lead to overall social transformation and progress in the society (Ajala, 2007; Bassey, 2011). Hence, the government is expected to play significant role in this regard especially in putting necessary structure and institution in place towards improving the education and socio-economic conditions of *Al-Majiri* students in the North. For instance, entrepreneurship skill and entrepreneurial activities can be introduced into system in order to make the learners to be self-independent after graduation (Bala, 2018). The leadership that will make an effective change for social transformation which would definitely put in place social institution whereby transparency and accountability shall be considered as its core values (Ajala, 2007).

It is paramount to note that, wellbeing of every citizen is primarily the responsibility of the government; however, collective cooperation of all citizens is required in this regard, in order to sustain the development and progress of the nation. Domestic policy for resource diversification is necessary for the improvement of social conditions of students whereby the government is expected to find solution to common problem facing *Al*-*Majiris* in many parts of the Northern Nigeria. This assertion is in consonance with the position of Daramola (2010) that, there is a great problem associated with development in all facets of human endeavours in the country.

## 7 Sustainable economic development among *Al-Majiri* children

Indeed, one of the foremost priorities of most political leaders in Nigeria is to achieve accelerated economic growth as literature contends (Bassey, 2011; Ogbo et al., 2017). Nonetheless, a number of factors serve as impediments to the dream of economic growth. Of such impediments are: unstable economic policies, political instability and corruption. Literature has explored the Nigerian economy and several economic policies and initiatives have been proposed to improve the economy (Akhakpe, 2009; Erhun, 2015). Nonetheless, street begging among *Al-Majiri* children in the Northern part of the country is an indicator of poverty despite the fact that the government has been trying to improve the economy. Nonetheless, the government has initiated different strategies that can bring meaningful economic growth in order to achieve a better society that will improve the conditions of the poor and more importantly, reduce poverty in a drastic way. There are several programmes in the country aims at reducing poverty and improving economic conditions of the citizens and of such programmes are: National Poverty Eradication Programme (NAPEP) (2001); National Economic Empowerments and Development Strategy (NEEDS) 2004). Literature contends that, NEEDS is a strategy for national economic development in order to attain sustainable development (Udunze et al., 2014). It is however noteworthy to say that despite the fact that the government's initiatives provide a framework for addressing poverty, but the strategies did not make significant impact in the lives of destitute *Al-Majiri* children in the Northern part of the country. The reason for ineffectiveness of the NEEDS is majorly corruption and bad leadership.

Onwards, sustainable economic development is an economic growth and development that attempts to improve the condition of citizens. In other words, sustainable economic development is a kind of economy that meet the needs of the citizens without affecting the lives of the future generation in responding to their needs (Kuhlman and Farrington, 2010). Thus, it is necessary to diversify the economy in order to achieve sustainable economic growth and development (Suberu et al., 2015) Based on the above submission, it can be inferred that, the current situation and condition of *Al-Majiri* children in the Northern part of the country. Thus, improvement of social economic conditions of *Al-Majiri* children is essential in order for government to address the problem of poverty in the country. In order to attain sustainable economy in the country, it is important to address the economic condition of destitute *Al-Majiri* children in the Northern part of the country. This is very significant in order to attain sustainability.

Furthermore, it is noteworthy to say that that, the essence of sustainable economy growth is to close the gap between the rich and the poor and more improve the economic condition of the poor. Negligence of solving the problem of poverty and street begging among *Al-Majiri* children in the North will consequently affect social orderliness and political stability. In other words, poverty indeed used to trigger violence. As a result, economic viability is necessary in attaining economic sustainability because it requires participation of stakeholders or decision makers, economic policy formulators and all sectors of economy in order to reduce the rate of poverty among the citizens as literature contends (Iwala, 2014; Erhun, 2015). Hence, introducing small scale business practices or entrepreneurship among *Al-Majiri* children will be instrumental in promoting the goals of sustainable economic development. It is important to incorporate vocational skills into *Al-Majiri* system in order to make them responsible human beings. Literature contends that, it is paramount that vocational education is incorporated into *Al-Majiri* schools in order to attain economic growth in the country (Bala, 2018). According to National Policy on Education (NPE), vocational and technical education is an aspect of education that make the learners acquire practical and applied skills. Thereby, *Al-Majiri* system should prepare students for skills in crafts, agriculture, industry, commerce and home economics. More specifically, vocational skills such as shoe-making, tailoring, mechanics, poultry farming, repair of phones, weaving among others will drastically reducing the rate of unemployment in the society. This is essential in order to address the challenge of joblessness among the graduate of Islamic oriented schools because it will not contribute to wealth creation and economic growth of the country specifically by increasing gross domestic product (GDP) (Imam, 2015; Bala, 2018). It is therefore vital that vocational skills are incorporated into the system so that, self-employment and self-reliance will be promoted among the students. More importantly, the *Al-Majiri* children can be used in developing local products that will lead to improvement of national economy in the country. Hence, cooperation is an essential aspect for the development and growth of competitive economy. The study by Ogbo et al. (2017) specifically notes that:

Long term viability of the local economies can be achieved and depends on the cooperation in a competitive economy, to avoid damaging the side effects of these competitions. A sustainable economy for a country, when considered, is all encompassing and cuts across all the facets of the economy. Economic sustainability forms an essential component of sustainable development; it is the achievement of development by maintaining and sustaining (p. 584).

The above quotation is very lucid with regards to the use of local economies as instrument for developing competitive economy. Nonetheless, the resources of the country cannot be underestimated, however, the country remains low in terms of financial performance especially in reducing high rate of poverty. Hence, the resources should be galvanized together in bringing about social, economic, educational, political, cultural, religious and environmental developments. It is on this note that the study by Dang and Sui Pheng (2015) noted that, infrastructural development is essential in fostering the economy. This is significant in the context of *Al-Majiri* system in the Northern part of the country.

## 8 Implications of the study

This paper contributes to the existing body of knowledge with specific emphasis on the inclusion of necessary skills that will make *Al-Majiri* children contribute to the overall socio-economic development in the country. In addition, the paper provides implications for Islamic education system, government, teachers, parents and society. Each of these is explained in the subsequent paragraphs.

First, the paper has implication on Islamic education system especially it will change the outlook of Islamic educational system from narrowed point of view to a holistic perspective especially by including subjects that will make the students contribute to the national development in the country. Second, the government has been advocating for a change agenda, in order to achieve meaningful change, it is paramount to initiate and implement economic policy that will improve the condition of destitute *Al-Majiri* children in the Northern part of the country. It is on this note that literature contends that, in order to achieve overall sustainable development in the country, it is important for the government to utilize holistic approach in improving social, economic and ecological dimensions in the country. The article identifies that the government has been making a lot of desired efforts in fostering *Al-Majiri* system through Universal Basic Education Commission (UBEC) and National Board for Arabic and Islamic Studies (NBAIS). It is however noted that, the government still need to more in the aspect of skill acquisition especially in the aspect of entrepreneurial activities in order to improve socio-economic condition of *Al-Majiri* and contribute immensely to the national progress and sustainable development in the country. Third, the teachers of *Almajiri* system will find this study useful especially they will be acquainted with the significance of their roles in cultivating economic development in *Al-Majiri* system. Fourth, the parents of children that are attending *Al-Majiri* system should encourage their children not to limit themselves to learning of religious subjects but they should also learn conventional subjects the AIMS has been promoting in order to foster socio-economic development for the attainment of national progress in the country. The society will significantly find the paper useful because the inclusion of conventional subjects into the curriculum of *Al-Majiri* system will drastically reduce the high level of poverty in the society. In so doing, the viable economic improvement of *Al-Majiri* children will bring about sustainable economic development as the literature posits that diversification of resource is paramount for sustainable economic development in the society.

## 9 Conclusion and suggestions

This paper has elaborated on the education for national progress and in consequentially achieving sustainable economic development. National Policy on Education has emphatically stressed on the vocational training in attaining economic growth. More importantly, there is lack of vocational training in *Al-Majiri* system, this paper has made it clear that, inclusion of vocational training will make *Al-Majiri* system to be functional and ontributive to sustainable economic development in the country. It has been emphatically noted that, the future of education specifically in *Al-Majiri* system depends on effective and efficient use of education for national advancement by harmonizing entrepreneurial activities into their programmes. This paper is important to other scholars that have been trying to address the problem of destitute and terrorism attributed to *Al-Majiri* children in the Northern part of the country. This has contributed to the existing literature with specific focus of making the system goes beyond teaching of religious subjects but there is an emphasis of on inclusion of vocational training and entrepreneurship skill. It has also been argued that, education is an instrument for sustainable economic development in the context of *Al-Majiri* system in the Northern part of the country.

Therefore, various suggestions for improvement of education in *Al-Majiri* system in the country are provided in order to achieve education for national progress and sustainable economic development. The effective measures for future educational efficiency depend largely on all the stakeholders namely: government, school administrators, parents, teachers, students, individuals and society. Each of these suggestions is presented as follows:

i. Government: The government through the activities of UBEC and NBAIS should jointly initiative skill acquisition centres specifically focusing on entrepreneurship skill and provide funds for *Al-Majiri* schools as an integral part of budget required for education as stipulated by the United Nations in order to make education reflects progress and socio-economic development in the country.
ii. School Administrators: The school management and proprietors of *Al-Majiri* schools should take effective and efficient steps for the implementation of government initiatives and improve the welfares of teachers in order to attain sustainable development in the country.
iii. Parents: There is need to create awareness among parents the knowledge acquisition in *Al-Majiri* schools especially with the newly introduced curriculum of NBAIS is not only limited to religious learning but it accommodates other subjects that can make them function well in the society and consequently contribute to overall development of the country.
iv. Teachers: As teachers occupy central climax in the implementation of educational curriculum and they play paramount roles in shaping the whole personality of the children. They are therefore expected to shape the learners holistically especially in informing that the vital importance of inclusion entrepreneurial activities in order to make education sustainable in *Al-Majiri* system.
v. Students: *Al-Majiri* Students are the pivot target specifically in preparing for educational progress and sustainable development. Hence, their orientation should change from street begging to acquisition of functional skills that will enable them contribute to general development of the society.
vi. Individuals: It is essential to note that individuals have role to play in raising the standard of living within the society especially by involving actively in socio-economic development of *Al-Majiri* system. In addition, the culture of maintenance should be revived which is an integral part of sustainability. Mismanagement or wastage of resources in the country immensely contributes to the backwardness the nation is experiencing in achieving sustainable development.
vii. Society: The entire society should give collective support and cooperation for the improvement of *Al-Majiri* system especially in laying foundation for adequate mechanism for the desired development of the whole society.

## Declarations

### Author contribution statement

Yusuff Jelili Amuda developed and the wrote this article.

### Funding statement

This work was supported by 10.13039/501100012639Prince Sultan University, RPF (1) 2020.

### Data availability statement

No data was used for the research described in the article.

### Declaration of interests statement

The authors declare no conflict of interest.

### Additional information

No additional information is available for this paper.
